# Susceptibility to mortality related to temperature and heat and cold wave duration in the population of Stockholm County, Sweden

**DOI:** 10.3402/gha.v7.22737

**Published:** 2014-03-12

**Authors:** Joacim Rocklöv, Bertil Forsberg, Kristie Ebi, Tom Bellander

**Affiliations:** 1 Department of Public Health and Clinical Medicine, Epidemiology and Global Health, Umeå University, Umeå, Sweden; 2 Department of Public Health and Clinical Medicine, Occupational and Environmental Medicine, Umeå University, Umeå, Sweden; 3 Institute of Environmental Medicine, Karolinska Institutet, Stockholm, Sweden; 4 Centre for Occupational and Environmental Medicine, Stockholm County Council, Stockholm, Sweden

**Keywords:** mortality, hospitalization, heat, cold, temperature, weather

## Abstract

**Background:**

Ambient temperatures can cause an increase in mortality. A better understanding is needed of how health status and other factors modify the risk associated with high and low temperatures, to improve the basis of preventive measures. Differences in susceptibility to temperature and to heat and cold wave duration are relatively unexplored.

**Objectives:**

We studied the associations between mortality and temperature and heat and cold wave duration, stratified by age and individual and medical factors.

**Methods:**

Deaths among all residents of Stockholm County between 1990 and 2002 were linked to discharge diagnosis data from hospital admissions, and associations were examined using the time stratified case-crossover design. Analyses were stratified by gender, age, pre-existing disease, country of origin, and municipality level wealth, and adjusted for potential confounding factors.

**Results:**

The effect on mortality by heat wave duration was higher for lower ages, in areas with lower wealth, for hospitalized patients younger than age 65. Odds were elevated among females younger than age 65, in groups with a previous hospital admission for mental disorders, and in persons with previous cardiovascular disease. Gradual increases in summer temperatures were associated with mortality in people older than 80 years, and with mortality in groups with a previous myocardial infarction and with chronic obstructive pulmonary disease (COPD) in the population younger than 65 years. During winter, mortality was associated with a decrease in temperature particularly in men and with the duration of cold spells for the population older than 80. A history of hospitalization for myocardial infarction increased the odds associated with cold temperatures among the population older than 65. Previous mental disease or substance abuse increased the odds of death among the population younger than 65.

**Conclusion:**

To increase effectiveness, we suggest preventive efforts should not assume susceptible groups are the same for warm and cold days and heat and cold waves, respectively.

Mortality related to high and low temperatures constitutes a large public health burden globally (1–4). In particular, large excess mortality has been associated with heat waves and cold spells (5–8), and recent studies have shown that mortality increases with the duration of such events over and above what would be predicted from a continuous temperature–mortality relationship (9–13). This may be explained by prolonged exposures being associated with physiological exhaustion related to cumulative stress over many consecutive days.

Studies of the impacts of weather and thermal extremes indicate that a number of health conditions and social factors may modify the mortality risk. A higher risk increase during heat waves was observed for individuals with pre-existing diabetes; heart disease; lung disease; mental illness and those using psychotropic medications; being obese; and those confined to bed (14–20). One study reported that hospitalization may be a protective factor during hot and cold episodes (15), while another study showed considerable increased risk of death (21). Studies of medical susceptibility factors for cold-related deaths are fewer, but indicate that the relative risk of death increases more in persons with cardiovascular dysfunction, as well as in persons with pre-existing chronic obstructive pulmonary disease (COPD) (15–17).

Other factors associated with modification of the risk of death during heat and heat waves include age, gender, clothing choices, use of cooling devices, and the socioeconomic situation (14, 16, 19, 20). Vigotti et al. reported that people born into and spending the first years of life in a warmer climate will be less susceptible to high temperatures compared to people born into and spending their youth in a colder climate (22).

For cold exposure, relative risks appear to be modified by age, gender, indoor temperatures, clothing behavior, and socioeconomic factors (16, 17, 23–25).

Although studies on susceptibility to temperatures are accumulating, no studies have studied and compared the groups susceptible to higher and lower temperature with groups susceptible to longer episodes of extreme temperature conditions (e.g. heat and cold wave duration).

## Objectives

We aim to study how daily temperature, and the duration of heat and cold waves, are associated with mortality, stratified by individual factors. Specifically, our objective is to describe the relationships between temperature and duration of heat and cold waves by groups of pre-existing diseases and previous hospital care, as well as by age, gender, country of birth, and wealth at municipal level.

## Material and methods

### The study location and population

Stockholm County, Sweden, is situated in northern Europe bordering the Baltic Sea. The study area has a cold temperate climate with summer temperatures (June–August) averaging 17°C and winter temperatures (December–February) averaging just below 0°C over the study period. The population in Stockholm County increased over the period 1990–2002, but stayed within the range of 1,600,000–1,800,000 throughout the study period. The increase was largely due to population movements; the fertility rate was stable. Life expectancy among women (above 80 years) was higher compared to men (below 80 years); both increased during the period of the study. The proportion of elderly people above the age of 65 was slightly smaller in Stockholm County compared to the nation overall, but amounted to nearly 20% of the study population.

### Subjects and individual information

We applied for and obtained mortality and hospitalization data for all residents in Stockholm County from the Swedish National Cause of Death Register (Swedish National Board of Health and Welfare) for the period 1990–2002 and matched it with the data on the same individual in the National Hospital Discharge Register (considered complete as from 1987), using anonymous numbers instead of social security numbers for record linkage. We created indicator variables denoting whether a subject was hospitalized on the day of death. The indicators of pre-existing conditions were based on underlying (main) or secondary contributing causes in the hospital discharge register. Hospitalizations 0–28 days before death were not considered as pre-existing disease in order to exclude the influence of a deterioration of the health status just before death. The causes of death and previous hospitalizations were classified using the International Statistical Classification of Diseases and Related Health Problems (ICD)-9 for the period 1990–1996 and ICD-10 for 1997 and onward. Deaths from external causes were not studied. For certain chronic conditions, we created indicators based on any previous hospitalization more than 28 days before death: diabetes mellitus (ICD-9: 250; ICD-10: E10-E14); COPD (ICD-9: 490–492, 494–496; ICD-10: J40, J44, J47, J67); and mental disease (ICD-9: 290–319; ICD-10: F). For other chronic diseases, for which patients were considered to be able to recover over time, we created indicators for hospitalization in a period 28 days to 2 years before death: acute myocardial infarction and recurrent myocardial infarction (ICD-9: 410; ICD-10: I21–I22); cardiovascular disease (ICD-9: 390–459; ICD-10: I); cerebrovascular disease (ICD-9: 430–438; ICD-10: I60–I69); respiratory disease (ICD-9: 460–519; ICD-10: J); and substance abuse (ICD-9: 303–305; ICD-10: F10-19).

The individuals’ municipalities of residency (*n*=21) were categorized according to average wealth per person, based on the wealth statistics for 2007 (Source: Statistics Sweden förmögenhetsstatistik). Three categories were created, using the approximate 20th and 80th percentiles as cutoff points. The individuals’ country of origin was categorized as Nordic (born in Sweden, Finland, Norway, or Denmark) and elsewhere.

The supplementary material includes the total mortality counts stratified by individual and medical factors for summer and winter periods (Supplementary file 1).

### Environmental variables

Meteorological and air pollution data were obtained from an urban background (roof-top) station in Central Stockholm, managed by the City of Stockholm Environment and Health Administration. The variables extracted included: daily mean temperature; daily maximum temperature; daily minimum temperature; daily mean levels of nitrogen oxides (NO_x_); and daytime maximum 8-hour mean ozone levels. Missing data were not imputed. Descriptive statistics for the air pollution and meteorological data are presented in Table 1.

**Table 1:** Descriptive statistics for daily environmental variables in Stockholm County, 1990–2002, per season

	June–Aug	Dec–Feb
Temperature (°C)	16.9±3.2 (8%)	−0.43±4.2 (6%)
Maximum temperature 2nd/98th percentile (°C)	27.1	−4.8
Minimum temperature 2nd/98th percentile (°C)	17.4	−9.8
NO_x_ (µg/m^3^)	23.0±12.0 (32%)	40.4±30.5 (31%)
Ozone (µg/m^3^)	57.2±15.2 (7%)	34.2±14.1 (31%)

Mean±standard deviation (proportion missing), range.

Based on previous studies, we constructed variables for lagged effects of exposure as averages over lag zero to one when studying the impact of summer temperatures, and for lags zero to six when studying the impact of winter temperatures (13, 26). To study additional effects occurring from the duration of temperature extremes (27) (the duration of heat and cold waves) we created a cold wave and a heat wave duration variable, considering days in sequence below and above the 2nd and 98th percentile of temperature, respectively. This variable was given the value 1 on the second day of the cold or heat wave, 2 on the third and so on. All other days, including the first day of a cold or heat wave, were given the value zero. As an example, the heat wave variable took on the values zero, zero, one, two, three, zero, and zero in a week if temperatures were below the 98th percentile on day one, above on days two through five, and below on the last two days. Both the heat duration variable and the cold duration variable can be interpreted as capturing lag interactive effects not described by distributed lag models, such as the lag strata employed here (27). For air pollution exposure variables (ozone and NO_x_), we constructed a running mean in lag strata zero to one, zero to six.

### Statistical analysis

Data were analyzed separately for the summer period, June–August, and the winter period, December–February, by establishing separate case-crossover models using the time-stratified design (28–30). The time stratified design compares the environmental condition, on an individual level, on the day of death with the environmental conditions on control days using the same weekdays in the same month in the same year as the death occurred for control days. The time-stratified design adjusts for time trends and weekday patterns by design. It also adjusts for confounding factors in the main effects, but not when studying effect modification comparing subgroups that may, for example, have different age structures. We therefore stratified analyses of modifying effects by age groups (less than 65, and 65 years and older) to avoid such bias. Additionally, we adjusted for calendar variables including national holidays and the day prior to the national holiday. More information on model specifications can be found in Supplementary file 2.

Based on prior studies, a linear description of the log relative risk of temperature was judged to sufficiently describe mortality (13, 31). However, to assure that any potential negative influence of low summer time temperatures was removed (as represented by a negative slope) on the estimated heat related slope, we employed a linear exposure–response relationship above the median maximum daily temperature for the summer period (20.7°C). In Supplementary file 3, we provide details on how the smooth versus the fitted linear function compared (Supplementary file 3). No threshold was employed when estimating the association of low temperatures in the winter months, but a linear association over the range of winter temperatures was fitted. In Supplementary file 4, we provide a sensitivity analysis of the linear assumption of heat and cold wave duration estimates. We identified the optimal models based on changing predictor variables of temperature and heat and cold wave duration using the Akaike Information Criterion (AIC). The results from different model runs with respect to AIC are provided in Supplementary file 5. The best model for the summer period used maximum temperature to describe risks related to heat intensity, and minimum temperature to describe risks related to heat wave duration (Supplementary file 5). The best model for the winter period used maximum temperature for describing both the effects of low temperatures and cold wave duration (Supplementary file 5).

Potential confounders (influenza deaths, NOx, and ozone) were included in the models if they were: 1) associated with the outcome variable based on *p*<0.1; and 2) changed the estimated relative effect by >10%. Influenza was adjusted for in all winter period models by including a variable of current level of influenza mortality in the population. However, air pollution variables did not fulfill the above criteria (Supplementary file 6).

We estimated the effect from temperature intensity (linear effect) and the additional effect from heat waves (duration variable; linear effect) simultaneously in the model. This was done to differentiate groups potentially susceptible to the different types of exposure. In Supplementary files 2 and 3, more details are provided on model specifications and on the influence of incorporating the two temperature variables for summer and winter in the fitted models. Duration of extreme events was considered to have potentially different mechanistic, environmental, and behavioral effects compared with increasing heat intensity overall. According to Spearman's correlation, the temperature variables and the duration variables had reasonably low correlation (0.35 in summer and −0.29 in winters), and the variance inflation factor (VIF) indicated no collinearity problems. A similar model (without individual susceptibility factors) was validated in a prior study (13) based on recommendations for model checking of the case-crossover model (32).

We present estimates as odds ratio in each of the groups studied per degree increase of temperature and per additional day of heat or cold wave duration. Odds ratios (OR's) are presented with corresponding 95% confidence intervals (CI).

## Results

**Fig. 1:**
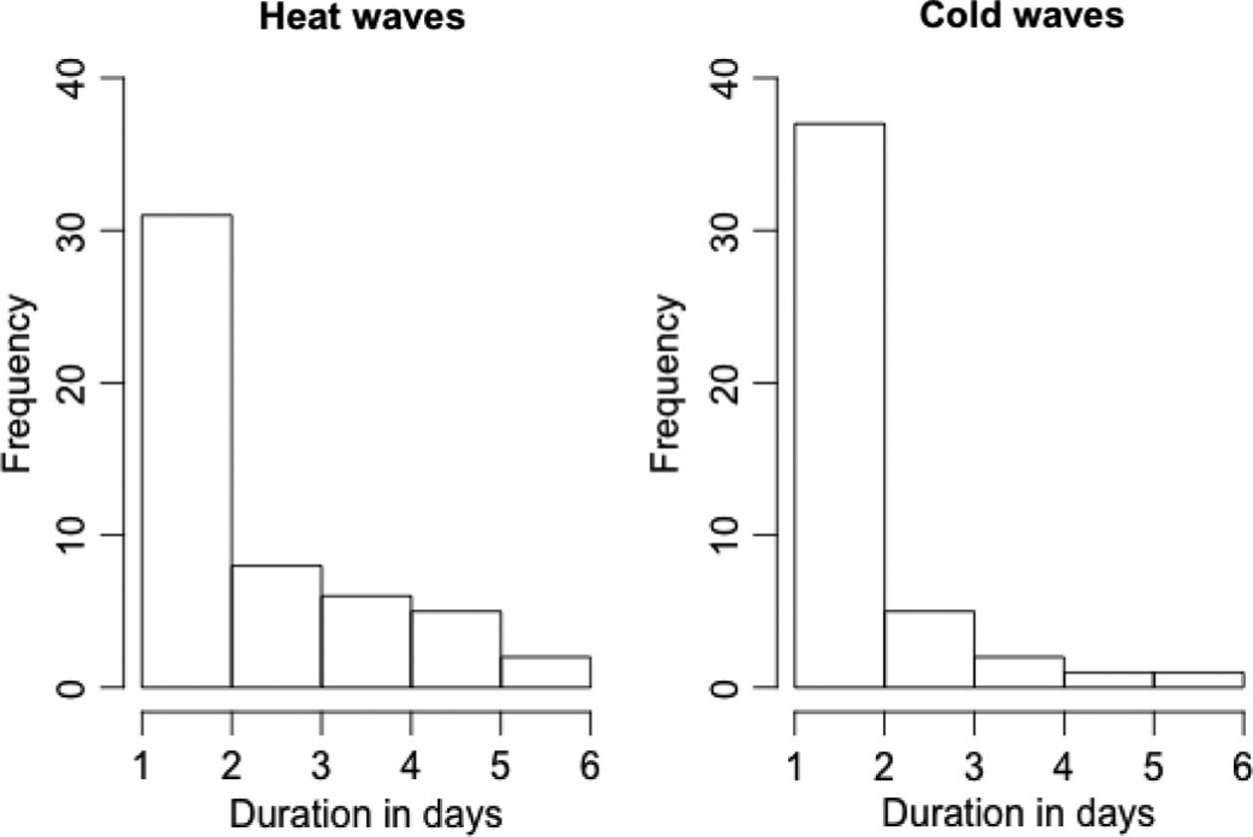
Cumulative frequency of occurrence of heat and cold wave duration events based on minimum daily temperature (17.4°C) in summer, and maximum daily temperatures (−4.8°C) in winter for the period 1990–2002 in Stockholm, Sweden.

Descriptive statistics for the environmental variables and mortality rates in Stockholm County, 1990–2002, are presented in Table 1. Cumulative frequencies of occurrence of heat wave and cold wave duration counts are described in Fig. 1. By cumulative frequency, we mean that each longer heat or cold wave also contributes to the frequency of the duration of shorter heat and cold waves in Fig. 1. The total deaths per strata of individual and medical factors are described in Supplementary file 1.

### High temperature and heat wave related estimates

**Fig. 2:**
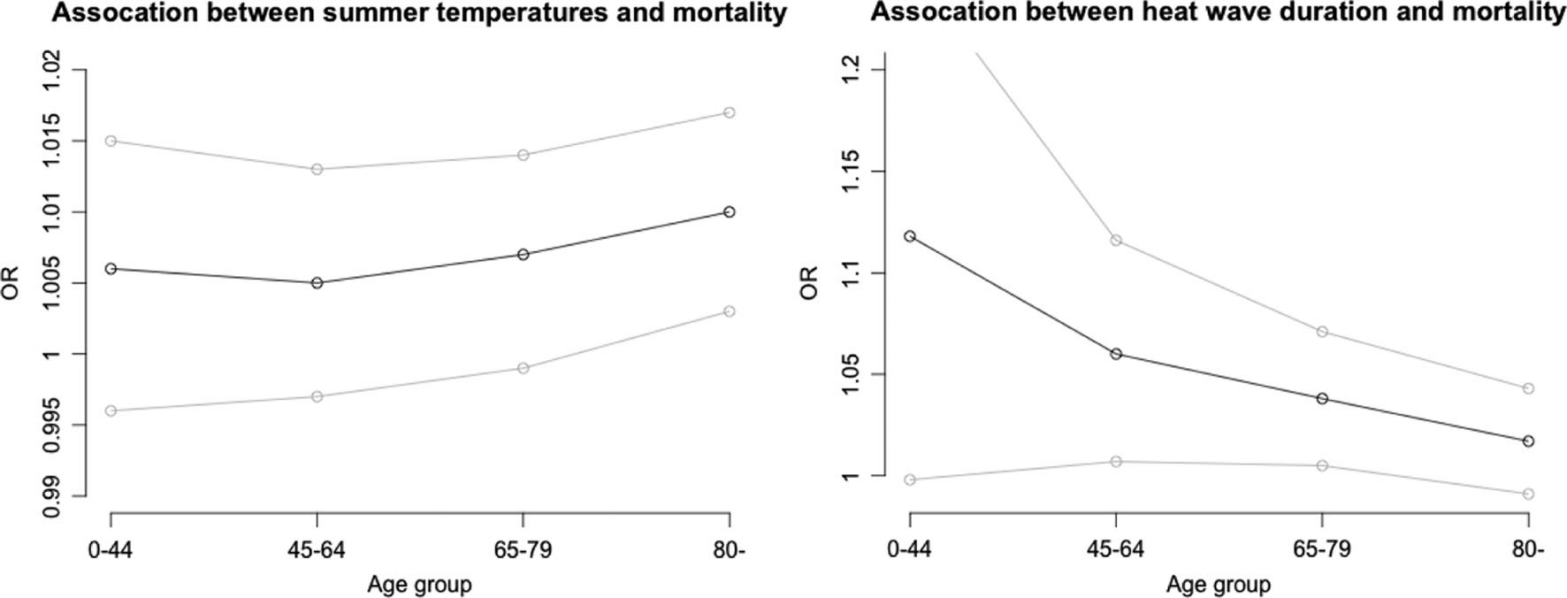
Age stratified odds ratios (black points) associated with linear increases in summer temperature (left), and heat wave duration (right). Grey point indicates the 95% confidence limits.

There was a significant increase in mortality risk in the population following increases in maximum temperature (Table 2). The heat wave duration variable, based on the daily minimum temperature (above 17.4°C), statistically and significantly affected mortality (Table 2). The heat wave duration association is larger in lower ages, compared with the effect of temperature, which only significantly affects mortality in people aged 80 and above (Fig. 2; Table 2).

**Table 2:** Odds ratios associated with summer temperature stratified by age and demographic factors with 95% confidence limits

Group	Odds ratio associated with a one unit increase of maximum temperature lag 0–1 (95% CI)	Odds ratio associated with heat wave duration (95% CI)
All population	**1.008 (1.001, 1.015)**	**1.031 (1.012, 1.051)**
Ages 0–44	1.006 (0.996, 1.015)	1.118 (0.998, 1.252)
Ages 45–64	1.005 (0.997, 1.013)	**1.060 (1.007, 1.116)**
Ages 65–79	1.007 (0.999, 1.014)	**1.038 (1.005, 1.071)**
Ages 80+	**1.010 (1.002, 1.017)**	1.017 (0.991, 1.043)
Men, age <65	1.004 (0.996, 1.012)	1.039 (0.976, 1.106)
Men, age≥65	**1.009 (1.001, 1.017)**	**1.046 (1.015, 1.078)**
Women, age<65	1.006 (0.997, 1.014)	**1.110 (1.035, 1.191)**
Women, age≥65	**1.007 (1.000, 1.015)**	1.009 (0.982, 1.036)
Low wealth, age<65	1.003 (0.993, 1.013)	**1.132 (1.019, 1.258)**
Low wealth, age≥65	**1.008** (1.000, 1.016)	1.000 (0.941, 1.063)
Medium wealth, age<65	1.001 (0.992, 1.010)	**1.086** (1.001, 1.178)
Medium wealth, age≥65	**1.009** (1.001, 1.017)	1.010 (0.970, 1.052)
High wealth, age<65	1.008 (0.999, 1.016)	1.037 (0.969, 1.109)
High wealth, age≥65	**1.008** (1.001, 1.016)	**1.034** (1.009, 1.060)
Born within the Nordic countries, age<65	1.005 (0.997, 1.013)	**1.076** (1.024, 1.131)
Born within the Nordic countries, age≥65	**1.008** (1.001, 1.016)	**1.023** (1.001, 1.045)
Born outside the Nordic countries, age<65	1.005 (0.995, 1.015)	1.015 (0.881, 1.169)
Born outside the Nordic countries, age≥65	1.007 (0.998, 1.015)	1.052 (0.976, 1.134)

Estimates with *p*<0.05 are marked as bold.

While men and women aged 65 or older appear to face similar risks to increases in temperature, men experience stronger impacts from longer heat wave duration for ages 65 and above, while women experience a large increase in mortality risks with heat wave duration in the age group below 65 (Table 2). The increase in women younger than age 65 is even more apparent when relaxing the linear assumption of the heat wave duration estimates (Supplementary file 4). Mortality in individuals living in low and medium wealth municipalities is not associated with temperature in ages 65 or above (Table 2). However, heat wave duration is associated with mortality in the populations younger than 65, with estimates increasing from an OR of 1.037 to a statistically significant 1.132 moving from the high wealth municipality to the low wealth municipality, respectively. Originating from a Nordic or non-Nordic country is generally not associated with large mortality differences, but the OR estimate in populations with Nordic origin showed a large and strong increase with heat wave duration (Table 2). However, the groups of those with non-Nordic origins are relatively small (Supplementary file 1).

Heat intensity is not associated with mortality in hospitalized patients, but is associated with mortality in non-hospitalized individuals over 65 (Table 3). Mortality in non-hospitalized patients younger than 65 significantly increases with heat wave duration, and even more so in hospitalized patients. Persons above 65 years with a pre-existing mental disorder are at dramatically increased odds of death with heat wave duration, and also with increasing temperatures in general (Table 3). Having pre-existing COPD or myocardial infarction is associated with large increases in death rates when temperature increases in persons younger than 65 (Table 3). In persons older than 65, mortality increases when temperature increases among persons with a pre-existing respiratory disease, and for heat wave duration among persons with a pre-existing cardiovascular disease (Table 3).

**Table 3:** Odds ratios associated with summer temperature stratified by age and medical factors with 95% confidence limits

Group	Odds ratio associated with a one unit increase of maximum temperature lag 0–1 (95% CI)	Odds ratio associated with heat wave duration (95% CI)
Out of hospital, age<65	1.005 (0.997, 1.013)	**1.059 (1.005, 1.115)**
Out of hospital, age≥65	**1.009 (1.001, 1.016)**	**1.024 (1.002, 1.047)**
Hospitalized, age<65	1.003 (0.993, 1.013)	**1.116 (1.004, 1.239)**
Hospitalized, age≥65	1.006 (0.998, 1.013)	1.029 (0.978, 1.083)
Hospitalization for diabetes mellitus, ever, age<65	1.009 (0.991, 1.026)	0.706 (0.473, 1.052)
Hospitalization for diabetes mellitus, ever, age≥65	1.005 (0.995, 1.014)	1.099 (0.991, 1.219)
Hospitalization for COPD, ever, age<65	**1.030 (1.003, 1.056)**	1.106 (0.821, 1.489)
Hospitalization for COPD, ever, age≥65	1.009 (0.998, 1.019)	1.099 (0.987, 1.222)
Hospitalization for mental disorder, ever, age<65	1.004 (0.990, 1.017)	1.054 (0.9032, 1.230)
Hospitalization for mental disorder, ever, age≥65	**1.011 (1.002, 1.019)**	**1.099 (1.027, 1.175)**
Hospitalization for substance use, ever, age<65	0.999 (0.984, 1.013)	0.970 (0.797, 1.178)
Hospitalization for substance use, ever, age≥65	0.996 (0.981, 1.010)	1.124 (0.922, 1.371)
Hospitalization for acute myocardial infarction within 28 days to 2 years, age<65	**1.041 (1.003, 1.082)**	0.343 (0.062, 1.883)
Hospitalization for acute myocardial infarction within 28 days to 2 years, age≥65	1.009 (0.9968, 1.021)	1.023 (0.969, 1.320)
Hospitalization for cerebrovascular disease within 28 days to 2 years, age<65	0.993 (0.964, 1.023)	1.098 (0.649, 1.857)
Hospitalization for cerebrovascular disease within 28 days to 2 years, age≥65	1.007 (0.997, 1.016)	1.056 (0.951, 1.173)
Hospitalization for cardiovascular disease within 28 days to 2 years<65	1.000 (0.994, 1.014)	1.009 (0.891, 1.142)
Hospitalization for cardiovascular disease within 28 days to 2 years≥65	1.006 (0.998, 1.014)	**1.067 (1.017, 1.117)**
Hospitalization for respiratory disease within 28 days to 2 years, age<65	1.002 (0.986, 1.017)	1.015 (0.827, 1.245)
Hospitalization for respiratory disease within 28 days to 2 years, age≥65	**1.009 (1.000, 1.018)**	1.047 (0.966, 1.133)

Estimates with *p*<0.05 are marked as bold.

### Low temperature and cold spell related estimates

The odds ratio of a one-degree decrease of maximum temperature on all causes of natural death is statistically significant, but did not reach significance when stratified into age groups (Table 4). The duration of cold waves (below −4.8°C) appear to mainly affect individuals older than 80. Men experienced higher increases in death rates compared with women when temperatures go down. The increase among women appeared more strongly related to cold wave duration (Table 4). Persons living in medium to high wealth municipalities experienced significant increases in mortality with successive colder temperatures among the populations above and below age 65, and with duration of cold waves in high wealth municipalities for ages 65 and above. People originating from the Nordic countries experience higher risks when temperature decreased compared with others (Table 4).

**Table 4:** Odds ratios associated with winter temperature stratified by age and demographic factors with 95% confidence limits

Group	Odds ratio associated with a one unit decrease of maximum temperature lag 0–6 (95% CI)	Odds ratio associated with cold wave duration (95% CI)
All population	**1.007 (1.002, 1.011)**	1.026 (0.997, 1.055)
Ages 0–44	1.017 (0.990, 1.046)	0.942 (0.782, 1.135)
Ages 45–64	1.010 (0.996, 1.023)	0.998 (0.916, 1.087)
Ages 65–79	1.006 (0.998, 1.014)	0.995 (0.945, 1.048)
Ages 80+	1.005 (0.999, 1.011)	**1.053 (1.013, 1.093)**
Men, age<65	1.015 (0.998, 1.030)	0.937 (0.843, 1.041)
Men, age≥65	**1.010 (1.002, 1.016)**	1.035 (0.987, 1.083)
Women, age<65	1.007 (0.988, 1.025)	1.055 (0.940, 1.182)
Women, age≥65	1.002 (0.996, 1.008)	1.028 (0.987, 1.071)
Low wealth, age<65	1.005 (0.976, 1.034)	0.885 (0.720, 1.088)
Low wealth, age≥65	1.000 (0.986, 1.013)	1.010 (0.919, 1.108)
Medium wealth, age<65	1.005 (0.983, 1.025)	1.047 (0.908, 1.207)
Medium wealth, age≥65	**1.018 (1.007, 1.027)**	1.009 (0.947, 1.073)
High wealth, age<65	**1.017 (1.000, 1.034)**	0.986 (0.888, 1.093)
High wealth, age≥65	1.002 (0.996, 1.008)	**1.043 (1.004, 1.083)**
Born within the Nordic countries, age<65	**1.015 (1.002, 1.028)**	0.969 (0.892, 1.053)
Born within the Nordic countries, age≥65	**1.008 (1.002, 1.012)**	1.031 (0.998, 1.065)
Born outside the Nordic countries, age<65	0.994 (0.966, 1.021)	1.112 (0.891, 1.387)
Born outside the Nordic countries, age≥65	0.994 (0.981, 1.007)	1.006 (0.898, 1.126)

Estimates with *p*<0.05 are marked as bold.

The strongest associations between low temperatures and mortality were seen in populations below age 65 with a background of substance abuse and admission for mental disease (Table 5). Significant associations of low temperatures with mortality are also seen in individuals with previous hospitalization for acute myocardial infarction, and in non-hospitalized populations aged 65 and above.

**Table 5:** Odds ratios associated with winter temperature stratified by age and medical factors with 95% confidence limits

Group	Odds ratio associated with a one unit decrease of maximum temperature lag 0–1 (95% CI)	Odds ratio associated with cold wave duration (95% CI)
Out of hospital, age<65	1.010 (0.996, 1.023)	0.985 (0.906, 1.071)
Out of hospital, age≥65	**1.007 (1.001, 1.012)**	1.024 (0.820, 1.242)
Hospitalized, age<65	1.018 (0.990, 1.047)	1.009 (0.990, 1.058)
Hospitalized, age≥65	0.998 (0.986, 1.010)	1.073 (0.992, 1.160)
Hospitalization for diabetes mellitus, ever, age<65	1.014 (0.951, 1.079)	0.855 (0.509, 1.435)
Hospitalization for diabetes mellitus, ever, age≥65	1.015 (0.990, 1.040)	0.892 (0.752, 1.060)
Hospitalization for COPD, ever, age<65	0.923 (0.839, 1.015)	1.370 (0.656, 2.863)
Hospitalization for COPD, ever, age≥65	1.023 (0.995, 1.051)	0.988 (0.804, 1.213)
Hospitalization for mental disorder, ever, age<65	**1.065 (1.019, 1.113)**	0.754 (0.534, 1.064)
Hospitalization for mental disorder, ever, age≥65	1.007 (0.989, 1.024)	1.010 (0.908, 1.122)
Hospitalization for substance use, ever, age<65	**1.059 (1.004, 1.117)**	0.783 (0.521, 1.173)
Hospitalization for substance use, ever, age≥65	0.989 (0.940, 1.040)	1.158 (0.868, 1.545)
Hospitalization for acute myocardial infarction within 28 days to 2 years, age<65	1.008 (0.858, 1.184)	1.165 (0.370, 3.668)
Hospitalization for acute myocardial infarction within 28 days to 2 years, age≥65	**1.043 (1.005, 1.082)**	0.981 (0.767, 1.254)
Hospitalization for cerebrovascular disease within 28 days to 2 years, age<65	0.936 (0.827, 1.059)	1.006 (0.418, 2.420)
Hospitalization for cerebrovascular disease within 28 days to 2 years, age≥65	1.015 (0.990, 1.042)	0.952 (0.796, 1.137)
Hospitalization for cardiovascular disease within 28 days to 2 years<65	1.009 (0.978, 1.040)	1.059 (0.929, 1.206)
Hospitalization for cardiovascular disease within 28 days to 2 years≥65	1.003 (0.991, 1.014)	1.031 (0.983, 1.080)
Hospitalization for respiratory disease within 28 days to 2 years, age<65	1.016 (0.958, 1.077)	1.020 (0.607, 1.583)
Hospitalization for respiratory disease within 28 days to 2 years, age≥65	1.008 (0.986, 1.029)	1.065 (0.925, 1.225)

COPD=chronic obstructive pulmonary disease. Estimates with a *p*<0.05 are marked as bold.

Non-age stratified estimates of demographic and medical factors can be found in Supplementary file 7.

## Discussions

We studied the association of mortality with hot and cold weather according to individual factors and history of previous in-hospital care. We separated the effects of temperature and heat wave and cold wave duration and found that the associations in the susceptible groups were modified by age when stratified differed according to the different types of exposure (intensity and duration). We found that mortality increased more in the elderly when temperature increases, while heat wave duration was strongly associated with increasing deaths rates with lower age. In particular, we found strong increases in mortality associated with heat wave duration among the populations less than age 65, with the largest risk increases in women and less wealthy municipalities. For successive temperature increases, the largest increases were seen for people with pre-existing COPD or myocardial infarction in the age group below 65. Among the population aged 65 or older, there were significant increases in mortality with increasing temperatures and with heat wave duration among persons with a mental health condition or a pre-existing cardiovascular disease. Decreasing temperatures in winter were more strongly associated with mortality in ages 65 and above for men, people living in wealthier municipalities, non-hospitalized populations, and persons with pre-existing myocardial infarction. The strongest associations in ages less than 65 were among people with a pre-condition of mental disease or substance abuse. Duration of cold waves appeared to affect the very elderly population the most.

A previous study in Stockholm also found a difference in heat and heat wave duration effects by age groups, while the increasing susceptibility to cold spell duration in the very elderly has not been shown before (13). The results support that women and men are susceptible to heat wave duration, though heat wave duration increased mortality in women <65 years whereas for men among 65+ years. The additional effects of gradually increasing temperatures mainly affect the very oldest population. Estimates from the 2003 heat wave in Europe indicated mortality increases in women were higher compared with men in both the elderly and younger populations (33). A key conclusion from our study is that both heat intensity and heat wave duration are important risk factors for temperature-related mortality; future research should include both components. Studies of temperature and mortality may miss significant susceptible groups (such as women <65 years) if they do not incorporate variables for the additional effects of heat wave length. The heat wave duration risks among non-elderly women may be associated with other factors contributing to higher risks, such as lower wealth and hospitalization, or to other susceptibility factors such as living on top floor, social isolation, and medication use (14, 18).

The large increases in death rates among individuals with pre-existing mental illness during hot temperatures and by duration of heat waves may partly be the result of heat interacting with prescription psychotropic medication (14), as well as the result of behavioral factors, risk perception interacting with medication and dehydration, and reduced thirst perception and ability to hydrate properly. In particular, because this association was strongest in the elderly population, it appears ageing-related mental disease, such as dementia, may explain a significant part of this association. Increasing death rates with cold in people with mental health issues and substance abuse conditions may relate to a reduced perception of health risks with substance use, as well as possibly less protection from cold exposure (34).

A pre-existing diabetic condition has been suggested to enhance the risks associated with heat (17, 19). This finding was not supported by our analysis. The results do support the overall association between heat (i.e. maximum temperature) and mortality for older persons with pre-existing respiratory disease and with heat wave duration for cardiovascular disease among the elderly (17–20, 35). Individuals having pre-existing COPD aged less than 65 experienced higher risks with elevated temperatures in summer. Prior studies found COPD weakly modified the mortality risk associated with cold (17). Hot weather can increase circulatory and cardiovascular strain by virtue of increases in deep body temperature or thermoregulatory processes (35), thus heat-related increases in COPD and cardiovascular mortality is perhaps unsurprising. However, the difference between age stratifications and COPD (mortality increasing in <65 years) and cardiovascular disease (mortality increasing in >65 years) is intriguing.

We estimate that most risk increases in daily mortality attributed to temperature occurred in non-hospitalized populations in both age groups. For heat, this is consistent with studies from the US (16, 36). However, a study from Italy reports that hospitalized individuals are susceptible to heat (21). Our results support this finding among hospitalized populations aged less than 65 during heat waves.

A study from Italy suggested that a warmer place of birth lowered the risk of heat-related mortality (22). Although our results did not exclude such an effect (in particular with heat wave duration this seems plausible), our results do not confirm the hypothesis that people with a northern origin also experience increased risks with cold conditions. A limitation of our study in this respect may be that populations from outside the Nordic countries (the variable used as a proxy for acclimatization to cold climate) also may be acclimatized to a cold climate.

Previous studies suggested that socioeconomic factors may or may not increase the risks with heat exposure (24, 37). A study by Anderson et al. indicated that the effects associated with heat waves were stronger in individuals with lower wealth (38). Our results support this finding, but also show that this association was related to heat wave duration and appeared strongest in people younger than 65. For low temperatures, it appeared that the wealthier populations might be at higher risk. This may be related to differences in the type of housing, insulation and heating between wealthy and less wealthy populations. For example, wealthier municipalities may be characterized to a larger extent by less energy efficient older houses.

This study presented the estimates per group of individual factors. While the estimates of susceptible groups to heat and cold according to pre-existing hospitalization have not been presented with a reference group estimate. The purpose for this is that a better reference to susceptibility appear the group estimates to each other instead of the group estimate to all other groups which would be the default reference category.

We only found some factors that appeared to predict larger risks to ambient high and low temperature conditions. However, we did not study factors related to activity level, social network, mobility, type of housing, and surrounding environment, which can affect temperature-related risk of death (18, 20).

The odds ratios assumed that the effects and effect-modifications were homogenous among age and sex groups. This may, however, not be the case. To study this with more detail, a much larger population or a longer time period would be necessary to have sufficient statistical power.

This study involved a large number of statistical tests, and may have resulted in some of the significant statistical tests being false positives.

The odds ratios related to weather were assumed to have the same linear shape and were estimated for the same thresholds for all sub-analyses, although this assumption was not extensively assessed; the supplementary material provides some sensitivity analyses. If such deviations exist, it might influence the estimates and so should be subject to a more thorough investigation. The risks related to high (and possibly low) temperatures might be modified by the synergistic effects of heat with simultaneously high levels of air pollution (39). However, we found a very small influence of air pollution, as shown in the supplementary files. If these effects appear only in certain conditions and as an additive interaction, then the initial adjustment in our study may have been inadequate. Unfortunately, we lacked a significant numbers of ozone observations and had no data on PM2.5. However, the air pollution measurements were not systematically missing with respect to temperature based on regression analysis (not reported). Further, studies in Stockholm over the same time period found negligent confounding with air pollution (1). Overall, studies show only small or no changes in effect estimates after simultaneously controlling for air pollution (33, 38).

## Conclusions

Temperature and the duration of extreme temperature episodes showed contrasting effects in population groups in Stockholm. In particular, heat exposure and heat wave duration showed different associations. Age, sex, hospitalization, and previous hospitalization for mental disorders or cardiovascular diseases were associated with stronger mortality effects of heat wave duration, especially among women, populations in less wealthy areas, and in hospital patients. Low temperatures were associated with stronger mortality effects in persons with previous hospitalization for acute myocardial infarction or mental disease, such as substance abuse in people younger than 65.

These findings need to be further explored to increase our understanding of which population groups experience increasing death rates with high and low temperature exposures, and with heat and cold wave duration. This information is important for the development of preventive actions to protect populations from the fatal effects of temperatures, including cold and heat waves. The results from this study provide valuable information for communicating to health care providers and those at particular risk in the general public during temperature extremes, and how those communications could be modified as extreme temperature events persist to reflect changing vulnerabilities.
